# Undeca­carbonyl-1κ^3^ *C*,2κ^4^ *C*,3κ^4^ *C*-(triethyl phosphite-1κ*P*)-*triangulo*-triruthenium(0)

**DOI:** 10.1107/S1600536811001450

**Published:** 2011-01-15

**Authors:** Omar bin Shawkataly, Mohd. Gulfam Alam, Chin Sing Yeap, Hoong-Kun Fun

**Affiliations:** a Chemical Sciences Programme, School of Distance Education, Universiti Sains Malaysia, 11800 USM, Penang, Malaysia; b X-ray Crystallography Unit, School of Physics, Universiti Sains Malaysia, 11800 USM, Penang, Malaysia

## Abstract

In the title *triangulo*-triruthenium compound, [Ru_3_(C_6_H_15_O_3_P)(CO)_11_], each Ru atom has distorted octa­hedral coord­ination geometry. The monodentate phosphine ligand is equatorially coordinated to one Ru atom, leaving one equatorial and two axial carbonyl substituents on the Ru atom. Each of the remaining two Ru atoms carries two equatorial and two axial carbonyl groups. In the crystal, mol­ecules are linked into an inversion dimer by a pair of inter­molecular C—H⋯O hydrogen bonds and the dimers are stacked along the *b* axis.

## Related literature

For related structures, see: Bruce *et al.* (1988 ▶); Churchill *et al.* (1977 ▶). For the synthesis, see: Bruce *et al.* (1987 ▶). For stability of the temperature controller used in data collection, see: Cosier & Glazer (1986 ▶).
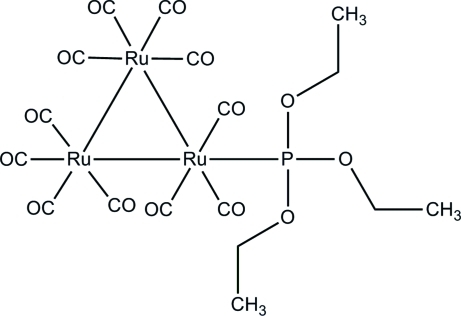


## Experimental

### 

#### Crystal data

[Ru_3_(C_6_H_15_O_3_P)(CO)_11_]
*M* *_r_* = 777.47
Monoclinic, 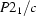

*a* = 12.8866 (3) Å
*b* = 9.0955 (2) Å
*c* = 21.7772 (5) Å
β = 99.589 (1)°
*V* = 2516.84 (10) Å^3^
*Z* = 4
Mo *K*α radiation
μ = 1.91 mm^−1^
*T* = 100 K
0.22 × 0.15 × 0.07 mm

#### Data collection

Bruker APEXII DUO CCD area-detector diffractometer
Absorption correction: multi-scan (*SADABS*; Bruker, 2009 ▶) *T*_min_ = 0.681, *T*_max_ = 0.880
48269 measured reflections
13224 independent reflections
10210 reflections with *I* > 2σ(*I*)
*R*_int_ = 0.049

#### Refinement

*R*[*F* ^2^ > 2σ(*F* ^2^)] = 0.033
*wR*(*F* ^2^) = 0.085
*S* = 1.05
13224 reflections
319 parameters
H-atom parameters constrained
Δρ_max_ = 1.01 e Å^−3^
Δρ_min_ = −1.23 e Å^−3^

### 

Data collection: *APEX2* (Bruker, 2009 ▶); cell refinement: *SAINT* (Bruker, 2009 ▶); data reduction: *SAINT*; program(s) used to solve structure: *SHELXTL* (Sheldrick, 2008 ▶); program(s) used to refine structure: *SHELXTL*; molecular graphics: *SHELXTL*; software used to prepare material for publication: *SHELXTL* and *PLATON* (Spek, 2009 ▶).

## Supplementary Material

Crystal structure: contains datablocks global, I. DOI: 10.1107/S1600536811001450/is2661sup1.cif

Structure factors: contains datablocks I. DOI: 10.1107/S1600536811001450/is2661Isup2.hkl

Additional supplementary materials:  crystallographic information; 3D view; checkCIF report

## Figures and Tables

**Table 1:** Hydrogen-bond geometry (Å, °)

*D*—H⋯*A*	*D*—H	H⋯*A*	*D*⋯*A*	*D*—H⋯*A*
C12—H12*A*⋯O2^i^	0.97	2.60	3.558 (3)	171

Symmetry code: (i) 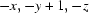.

**Fig. 1.**
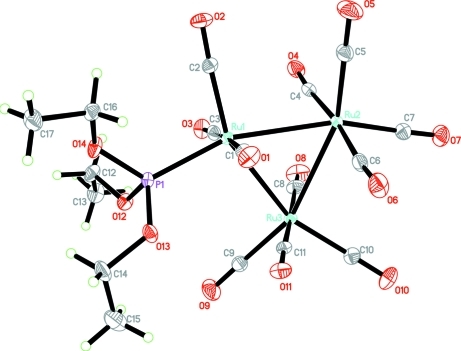
The molecular structure of (I) with 50% probability ellipsoids for non-H atoms.

**Fig. 2.**
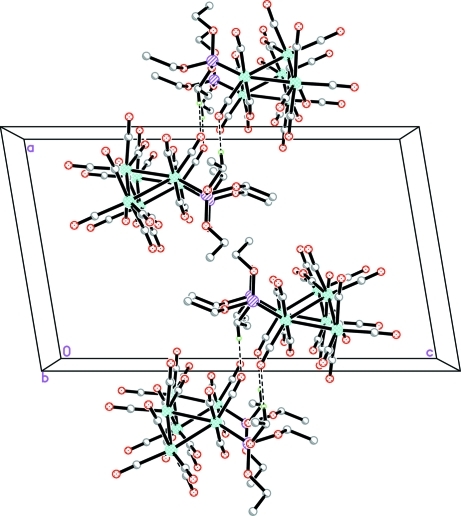
The crystal packing of (I), viewed down the b axis, showing the molecules are linked into dimers. Hydrogen atoms not involved in the hydrogen-bonding (dashed lines) have been omitted for clarity.

## supplementary crystallographic information

### Comment

Syntheses and crystallographic structures of substituted *triangulo*-triruthenium clusters have been of interest to researchers due to structural variation and catalytic activity. As part of our ongoing studies on phosphine substituted *triangulo*-triruthenium clusters, herein we report the structure of title compound, (I).

In the title compound, the monodantate phosphine ligand has replaced a carbonyl group in the equatorial plane of the Ru_3_ triangle. The *triangulo*-triruthenium is bonded equatorially to a monodentate phosphine ligand. The Ru2—Ru3 bond is noticeably longer [2.8605 (2) Å] compared to the other two Ru—Ru bonds [2.8348 (2) and 2.8436 (2) Å]. The unusual increase in the length of Ru—Ru bond in comparison with those of Ru_3_(CO)_12_ structure (Churchill *et al.*, 1977) can be attributed to the steric effect induced by the bulky substituent.

As observed in Ru_3_(CO)_12_, the bond from metal atoms to the axial CO groups in complex (I) are longer (Ru—C_ave_ = 1.941 Å) compared to the equatorial CO groups (Ru—C_ave_ = 1.917 Å). The equatorial Ru—C—O moieties are linear (average angle: 178.30°) whereas the axial Ru—C—O moieties are slightly bent (average angle: 174.14°). Similar observations were made by Bruce and co-workers for the range of monosubstituted complexes synthesized by them (Bruce *et al.*, 1988).

In the crystal structure, the molecules are linked into dimers by intermolecular C12—H12A···O2 hydrogen bonds and stacked down the *b* axis (Fig. 2 and Table 1).

### Experimental

All the manipulations were performed under a dry oxygen-free nitrogen atmosphere using standard Shlenk technique. THF was dried over sodium wire and freshly distilled from sodium benzophenone ketyl solution. Radical anion method was used for the synthesis of complex (Bruce *et al.*, 1987). The title compound (I) was prepared by mixing Ru_3_(CO)_12_ (Aldrich) and P(O–CH_2_CH_3_)_3_ (BDH Chemicals Ltd. Poole England) in a 1:1 molar ratio in THF at 313 K. Separation of the product in pure form was done by column chromatography (Florisil, 100–200 mesh, eluant, dicholomethane: hexane). IR (cyclohexane): γ (CO) 2100, 2045, 2031 and 2015 cm^-1^. Crystals suitable for X-ray diffraction were grown from dichlomethane/methanol solution at 283 K.

### Refinement

All hydrogen atoms were positioned geometrically and refined using a riding model, with C—H = 0.93–0.97 Å and *U*_iso_(H) = 1.2 or 1.5*U*_eq_(C). The maximum and minimum residual electron density peaks of 1.01 and -1.23 e Å^-3^ were located 0.74 and 0.69 Å from atoms C12 and Ru3, respectively.

### Crystal data

**Table:**

[Ru_3_(C_6_H_15_O_3_P)(CO)_11_]	*F*(000) = 1504
*M**_r_* = 777.47	*D*_x_ = 2.052 Mg m^−^^3^
Monoclinic, *P*2_1_/*c*	Mo *K*α radiation, λ = 0.71073 Å
Hall symbol: -P 2ybc	Cell parameters from 9999 reflections
*a* = 12.8866 (3) Å	θ = 2.8–37.4°
*b* = 9.0955 (2) Å	µ = 1.91 mm^−^^1^
*c* = 21.7772 (5) Å	*T* = 100 K
β = 99.589 (1)°	Plate, orange
*V* = 2516.84 (10) Å^3^	0.22 × 0.15 × 0.07 mm
*Z* = 4	

### Data collection

**Table:**

Bruker APEXII DUO CCD area-detector diffractometer	13224 independent reflections
Radiation source: fine-focus sealed tube	10210 reflections with *I* > 2σ(*I*)
graphite	*R*_int_ = 0.049
φ and ω scans	θ_max_ = 37.6°, θ_min_ = 2.3°
Absorption correction: multi-scan (*SADABS*; Bruker, 2009)	*h* = −22→22
*T*_min_ = 0.681, *T*_max_ = 0.880	*k* = −14→15
48269 measured reflections	*l* = −37→36

### Refinement

**Table:**

Refinement on *F*^2^	Primary atom site location: structure-invariant direct methods
Least-squares matrix: full	Secondary atom site location: difference Fourier map
*R*[*F*^2^ > 2σ(*F*^2^)] = 0.033	Hydrogen site location: inferred from neighbouring sites
*wR*(*F*^2^) = 0.085	H-atom parameters constrained
*S* = 1.05	*w* = 1/[σ^2^(*F*_o_^2^) + (0.0375*P*)^2^ + 0.1586*P*] where *P* = (*F*_o_^2^ + 2*F*_c_^2^)/3
13224 reflections	(Δ/σ)_max_ = 0.001
319 parameters	Δρ_max_ = 1.01 e Å^−^^3^
0 restraints	Δρ_min_ = −1.23 e Å^−^^3^

### Special details

**Table:**

Experimental. The crystal was placed in the cold stream of an Oxford Cryosystems Cobra open-flow nitrogen cryostat (Cosier & Glazer, 1986) operating at 100.0 (1) K.
Geometry. All e.s.d.'s (except the e.s.d. in the dihedral angle between two l.s. planes) are estimated using the full covariance matrix. The cell e.s.d.'s are taken into account individually in the estimation of e.s.d.'s in distances, angles and torsion angles; correlations between e.s.d.'s in cell parameters are only used when they are defined by crystal symmetry. An approximate (isotropic) treatment of cell e.s.d.'s is used for estimating e.s.d.'s involving l.s. planes.
Refinement. Refinement of *F*^2^ against ALL reflections. The weighted *R*-factor *wR* and goodness of fit *S* are based on *F*^2^, conventional *R*-factors *R* are based on *F*, with *F* set to zero for negative *F*^2^. The threshold expression of *F*^2^ > σ(*F*^2^) is used only for calculating *R*-factors(gt) *etc*. and is not relevant to the choice of reflections for refinement. *R*-factors based on *F*^2^ are statistically about twice as large as those based on *F*, and *R*- factors based on ALL data will be even larger.

### Fractional atomic coordinates and isotropic or equivalent isotropic displacement parameters (Å^2^)

**Table:**

	*x*	*y*	*z*	*U*_iso_*/*U*_eq_	
Ru1	0.206479 (12)	0.525270 (18)	0.104476 (7)	0.01058 (3)	
Ru2	0.171824 (13)	0.403007 (18)	0.219080 (7)	0.01213 (4)	
Ru3	0.307938 (12)	0.651191 (18)	0.218302 (7)	0.01164 (4)	
P1	0.28271 (4)	0.65310 (6)	0.03363 (2)	0.01287 (9)	
O1	0.36303 (14)	0.2725 (2)	0.09811 (8)	0.0216 (3)	
O2	0.03380 (14)	0.3498 (2)	0.02588 (9)	0.0236 (4)	
O3	0.06213 (14)	0.7945 (2)	0.10448 (8)	0.0226 (3)	
O4	−0.03552 (14)	0.5757 (2)	0.18670 (9)	0.0234 (3)	
O5	0.05504 (19)	0.1252 (2)	0.16827 (11)	0.0359 (5)	
O6	0.38235 (15)	0.2370 (2)	0.24542 (10)	0.0292 (4)	
O7	0.15360 (16)	0.3907 (2)	0.35704 (9)	0.0281 (4)	
O8	0.11480 (15)	0.8168 (2)	0.24884 (10)	0.0301 (4)	
O9	0.39514 (19)	0.9359 (2)	0.17244 (10)	0.0331 (5)	
O10	0.40533 (17)	0.6511 (2)	0.35681 (9)	0.0302 (4)	
O11	0.49842 (13)	0.4816 (2)	0.18586 (8)	0.0208 (3)	
O12	0.26748 (14)	0.82648 (18)	0.02947 (8)	0.0176 (3)	
O13	0.40697 (13)	0.63666 (19)	0.04805 (8)	0.0186 (3)	
O14	0.24886 (15)	0.61629 (18)	−0.03883 (7)	0.0194 (3)	
C1	0.30697 (17)	0.3662 (2)	0.10385 (10)	0.0147 (4)	
C2	0.09977 (17)	0.4158 (2)	0.05380 (10)	0.0155 (4)	
C3	0.11634 (17)	0.6941 (2)	0.10820 (10)	0.0156 (4)	
C4	0.04387 (17)	0.5178 (3)	0.19786 (10)	0.0168 (4)	
C5	0.0981 (2)	0.2289 (3)	0.18692 (11)	0.0207 (4)	
C6	0.30722 (19)	0.3036 (3)	0.23406 (11)	0.0193 (4)	
C7	0.16199 (19)	0.3959 (3)	0.30638 (11)	0.0190 (4)	
C8	0.18282 (19)	0.7500 (3)	0.23584 (11)	0.0196 (4)	
C9	0.36313 (19)	0.8289 (3)	0.18873 (11)	0.0201 (4)	
C10	0.36806 (19)	0.6518 (3)	0.30534 (11)	0.0193 (4)	
C11	0.42440 (17)	0.5392 (2)	0.19592 (10)	0.0153 (4)	
C12	0.1837 (2)	0.8957 (3)	−0.01318 (11)	0.0199 (4)	
H12A	0.1198	0.8381	−0.0159	0.024*	
H12B	0.2027	0.9010	−0.0544	0.024*	
C13	0.1655 (2)	1.0469 (3)	0.00963 (12)	0.0241 (5)	
H13A	0.1096	1.0930	−0.0184	0.036*	
H13B	0.2287	1.1040	0.0115	0.036*	
H13C	0.1467	1.0410	0.0504	0.036*	
C14	0.4751 (2)	0.7164 (3)	0.01137 (12)	0.0246 (5)	
H14A	0.4326	0.7588	−0.0253	0.029*	
H14B	0.5248	0.6486	−0.0023	0.029*	
C15	0.5331 (2)	0.8348 (3)	0.04981 (16)	0.0331 (6)	
H15A	0.5808	0.8820	0.0266	0.050*	
H15B	0.5721	0.7930	0.0872	0.050*	
H15C	0.4840	0.9058	0.0605	0.050*	
C16	0.2421 (2)	0.4681 (3)	−0.06278 (10)	0.0187 (4)	
H16A	0.1694	0.4357	−0.0706	0.022*	
H16B	0.2820	0.4018	−0.0329	0.022*	
C17	0.2865 (2)	0.4684 (3)	−0.12234 (12)	0.0249 (5)	
H17A	0.2852	0.3703	−0.1387	0.037*	
H17B	0.3577	0.5035	−0.1143	0.037*	
H17C	0.2449	0.5317	−0.1521	0.037*	

### Atomic displacement parameters (Å^2^)

**Table:**

	*U*^11^	*U*^22^	*U*^33^	*U*^12^	*U*^13^	*U*^23^
Ru1	0.01067 (6)	0.01134 (7)	0.00955 (6)	−0.00004 (5)	0.00112 (5)	−0.00073 (5)
Ru2	0.01260 (7)	0.01192 (7)	0.01229 (7)	−0.00088 (5)	0.00334 (5)	0.00087 (5)
Ru3	0.01155 (7)	0.01239 (7)	0.01087 (6)	−0.00163 (5)	0.00152 (5)	−0.00204 (5)
P1	0.0155 (2)	0.0126 (2)	0.0107 (2)	0.00183 (18)	0.00276 (17)	0.00072 (17)
O1	0.0209 (8)	0.0210 (8)	0.0220 (8)	0.0069 (6)	0.0007 (6)	−0.0032 (7)
O2	0.0208 (8)	0.0180 (8)	0.0288 (9)	−0.0028 (6)	−0.0056 (7)	−0.0038 (7)
O3	0.0234 (8)	0.0226 (9)	0.0229 (8)	0.0073 (7)	0.0066 (7)	0.0014 (7)
O4	0.0166 (7)	0.0254 (9)	0.0290 (9)	0.0012 (7)	0.0062 (7)	0.0055 (7)
O5	0.0462 (13)	0.0249 (10)	0.0380 (11)	−0.0166 (9)	0.0112 (10)	−0.0098 (9)
O6	0.0238 (9)	0.0294 (10)	0.0353 (10)	0.0089 (8)	0.0075 (8)	0.0125 (8)
O7	0.0357 (11)	0.0326 (10)	0.0172 (8)	−0.0016 (8)	0.0075 (7)	−0.0009 (7)
O8	0.0245 (9)	0.0294 (10)	0.0372 (11)	0.0040 (8)	0.0076 (8)	−0.0160 (8)
O9	0.0475 (13)	0.0230 (9)	0.0294 (10)	−0.0131 (9)	0.0081 (9)	0.0009 (8)
O10	0.0352 (11)	0.0374 (11)	0.0158 (8)	0.0047 (9)	−0.0026 (7)	−0.0027 (8)
O11	0.0152 (7)	0.0235 (8)	0.0233 (8)	−0.0009 (6)	0.0018 (6)	−0.0056 (7)
O12	0.0223 (8)	0.0123 (7)	0.0173 (7)	0.0014 (6)	0.0005 (6)	0.0005 (5)
O13	0.0146 (7)	0.0231 (8)	0.0195 (7)	0.0038 (6)	0.0064 (6)	0.0077 (6)
O14	0.0314 (9)	0.0153 (7)	0.0109 (6)	0.0033 (6)	0.0015 (6)	−0.0005 (5)
C1	0.0141 (8)	0.0164 (9)	0.0130 (8)	−0.0016 (7)	0.0004 (7)	−0.0001 (7)
C2	0.0157 (9)	0.0141 (9)	0.0159 (8)	0.0019 (7)	0.0003 (7)	0.0002 (7)
C3	0.0169 (9)	0.0171 (9)	0.0140 (8)	−0.0007 (7)	0.0058 (7)	−0.0011 (7)
C4	0.0154 (9)	0.0176 (10)	0.0177 (9)	−0.0034 (8)	0.0041 (7)	0.0015 (8)
C5	0.0246 (11)	0.0203 (10)	0.0180 (9)	−0.0028 (9)	0.0062 (8)	−0.0013 (8)
C6	0.0196 (10)	0.0185 (10)	0.0202 (10)	0.0001 (8)	0.0048 (8)	0.0055 (8)
C7	0.0200 (10)	0.0180 (10)	0.0191 (9)	−0.0001 (8)	0.0042 (8)	−0.0003 (8)
C8	0.0199 (10)	0.0203 (11)	0.0181 (9)	−0.0018 (8)	0.0021 (8)	−0.0075 (8)
C9	0.0229 (11)	0.0208 (11)	0.0164 (9)	−0.0042 (8)	0.0025 (8)	−0.0030 (8)
C10	0.0207 (10)	0.0206 (11)	0.0169 (9)	0.0005 (8)	0.0043 (8)	−0.0033 (8)
C11	0.0143 (8)	0.0175 (9)	0.0135 (8)	−0.0040 (7)	0.0002 (7)	−0.0016 (7)
C12	0.0238 (11)	0.0162 (10)	0.0175 (9)	0.0046 (8)	−0.0033 (8)	0.0022 (8)
C13	0.0307 (13)	0.0167 (10)	0.0248 (11)	0.0060 (9)	0.0041 (9)	0.0006 (9)
C14	0.0213 (11)	0.0305 (13)	0.0247 (11)	0.0007 (10)	0.0120 (9)	0.0073 (10)
C15	0.0271 (13)	0.0315 (15)	0.0424 (16)	−0.0030 (11)	0.0109 (12)	0.0071 (12)
C16	0.0248 (11)	0.0177 (10)	0.0138 (8)	−0.0025 (8)	0.0036 (8)	−0.0032 (7)
C17	0.0323 (13)	0.0234 (12)	0.0222 (10)	−0.0039 (10)	0.0134 (9)	−0.0061 (9)

### Geometric parameters (Å, °)

**Table:**

Ru1—C2	1.896 (2)	O8—C8	1.140 (3)
Ru1—C3	1.935 (2)	O9—C9	1.136 (3)
Ru1—C1	1.943 (2)	O10—C10	1.144 (3)
Ru1—P1	2.2808 (6)	O11—C11	1.141 (3)
Ru1—Ru2	2.8348 (2)	O12—C12	1.446 (3)
Ru1—Ru3	2.8436 (2)	O13—C14	1.472 (3)
Ru2—C5	1.918 (2)	O14—C16	1.443 (3)
Ru2—C7	1.927 (2)	C12—C13	1.494 (3)
Ru2—C4	1.941 (2)	C12—H12A	0.9700
Ru2—C6	1.944 (2)	C12—H12B	0.9700
Ru2—Ru3	2.8605 (2)	C13—H13A	0.9600
Ru3—C9	1.920 (2)	C13—H13B	0.9600
Ru3—C10	1.925 (2)	C13—H13C	0.9600
Ru3—C8	1.939 (2)	C14—C15	1.487 (4)
Ru3—C11	1.942 (2)	C14—H14A	0.9700
P1—O13	1.5867 (17)	C14—H14B	0.9700
P1—O12	1.5899 (17)	C15—H15A	0.9600
P1—O14	1.6011 (17)	C15—H15B	0.9600
O1—C1	1.137 (3)	C15—H15C	0.9600
O2—C2	1.132 (3)	C16—C17	1.502 (3)
O3—C3	1.144 (3)	C16—H16A	0.9700
O4—C4	1.140 (3)	C16—H16B	0.9700
O5—C5	1.134 (3)	C17—H17A	0.9600
O6—C6	1.134 (3)	C17—H17B	0.9600
O7—C7	1.127 (3)	C17—H17C	0.9600
			
C2—Ru1—C3	93.38 (9)	C12—O12—P1	122.72 (15)
C2—Ru1—C1	91.40 (9)	C14—O13—P1	121.31 (15)
C3—Ru1—C1	175.12 (9)	C16—O14—P1	122.81 (14)
C2—Ru1—P1	103.08 (7)	O1—C1—Ru1	174.12 (19)
C3—Ru1—P1	87.31 (7)	O2—C2—Ru1	176.8 (2)
C1—Ru1—P1	90.70 (7)	O3—C3—Ru1	173.60 (19)
C2—Ru1—Ru2	95.80 (7)	O4—C4—Ru2	174.5 (2)
C3—Ru1—Ru2	95.20 (6)	O5—C5—Ru2	179.4 (3)
C1—Ru1—Ru2	85.24 (6)	O6—C6—Ru2	174.3 (2)
P1—Ru1—Ru2	160.779 (16)	O7—C7—Ru2	178.2 (2)
C2—Ru1—Ru3	154.49 (7)	O8—C8—Ru3	174.2 (2)
C3—Ru1—Ru3	80.55 (6)	O9—C9—Ru3	178.3 (2)
C1—Ru1—Ru3	95.50 (6)	O10—C10—Ru3	178.8 (2)
P1—Ru1—Ru3	101.362 (15)	O11—C11—Ru3	174.12 (19)
Ru2—Ru1—Ru3	60.497 (6)	O12—C12—C13	109.20 (19)
C5—Ru2—C7	102.86 (10)	O12—C12—H12A	109.8
C5—Ru2—C4	90.43 (10)	C13—C12—H12A	109.8
C7—Ru2—C4	93.44 (10)	O12—C12—H12B	109.8
C5—Ru2—C6	93.06 (11)	C13—C12—H12B	109.8
C7—Ru2—C6	91.35 (10)	H12A—C12—H12B	108.3
C4—Ru2—C6	173.31 (10)	C12—C13—H13A	109.5
C5—Ru2—Ru1	98.25 (7)	C12—C13—H13B	109.5
C7—Ru2—Ru1	158.04 (7)	H13A—C13—H13B	109.5
C4—Ru2—Ru1	80.31 (7)	C12—C13—H13C	109.5
C6—Ru2—Ru1	93.53 (7)	H13A—C13—H13C	109.5
C5—Ru2—Ru3	156.39 (7)	H13B—C13—H13C	109.5
C7—Ru2—Ru3	99.99 (7)	O13—C14—C15	109.9 (2)
C4—Ru2—Ru3	94.11 (7)	O13—C14—H14A	109.7
C6—Ru2—Ru3	80.44 (7)	C15—C14—H14A	109.7
Ru1—Ru2—Ru3	59.903 (6)	O13—C14—H14B	109.7
C9—Ru3—C10	102.53 (10)	C15—C14—H14B	109.7
C9—Ru3—C8	92.44 (11)	H14A—C14—H14B	108.2
C10—Ru3—C8	90.93 (10)	C14—C15—H15A	109.5
C9—Ru3—C11	90.71 (10)	C14—C15—H15B	109.5
C10—Ru3—C11	92.73 (9)	H15A—C15—H15B	109.5
C8—Ru3—C11	174.55 (9)	C14—C15—H15C	109.5
C9—Ru3—Ru1	100.89 (7)	H15A—C15—H15C	109.5
C10—Ru3—Ru1	155.64 (7)	H15B—C15—H15C	109.5
C8—Ru3—Ru1	94.69 (7)	O14—C16—C17	107.56 (19)
C11—Ru3—Ru1	80.35 (6)	O14—C16—H16A	110.2
C9—Ru3—Ru2	158.16 (7)	C17—C16—H16A	110.2
C10—Ru3—Ru2	98.23 (7)	O14—C16—H16B	110.2
C8—Ru3—Ru2	80.43 (7)	C17—C16—H16B	110.2
C11—Ru3—Ru2	95.04 (6)	H16A—C16—H16B	108.5
Ru1—Ru3—Ru2	59.600 (6)	C16—C17—H17A	109.5
O13—P1—O12	102.46 (9)	C16—C17—H17B	109.5
O13—P1—O14	106.00 (10)	H17A—C17—H17B	109.5
O12—P1—O14	98.07 (9)	C16—C17—H17C	109.5
O13—P1—Ru1	110.63 (6)	H17A—C17—H17C	109.5
O12—P1—Ru1	118.77 (7)	H17B—C17—H17C	109.5
O14—P1—Ru1	118.86 (7)		
			
C2—Ru1—Ru2—C5	19.56 (10)	C4—Ru2—Ru3—C9	−47.3 (2)
C3—Ru1—Ru2—C5	113.51 (10)	C6—Ru2—Ru3—C9	128.8 (2)
C1—Ru1—Ru2—C5	−71.37 (10)	Ru1—Ru2—Ru3—C9	29.0 (2)
P1—Ru1—Ru2—C5	−149.73 (9)	C5—Ru2—Ru3—C10	−145.0 (2)
Ru3—Ru1—Ru2—C5	−170.44 (8)	C7—Ru2—Ru3—C10	20.26 (10)
C2—Ru1—Ru2—C7	−144.3 (2)	C4—Ru2—Ru3—C10	114.49 (10)
C3—Ru1—Ru2—C7	−50.4 (2)	C6—Ru2—Ru3—C10	−69.43 (10)
C1—Ru1—Ru2—C7	124.7 (2)	Ru1—Ru2—Ru3—C10	−169.20 (7)
P1—Ru1—Ru2—C7	46.4 (2)	C5—Ru2—Ru3—C8	125.5 (2)
Ru3—Ru1—Ru2—C7	25.66 (19)	C7—Ru2—Ru3—C8	−69.29 (10)
C2—Ru1—Ru2—C4	−69.46 (9)	C4—Ru2—Ru3—C8	24.94 (10)
C3—Ru1—Ru2—C4	24.49 (9)	C6—Ru2—Ru3—C8	−158.98 (10)
C1—Ru1—Ru2—C4	−160.39 (9)	Ru1—Ru2—Ru3—C8	101.24 (7)
P1—Ru1—Ru2—C4	121.25 (8)	C5—Ru2—Ru3—C11	−51.5 (2)
Ru3—Ru1—Ru2—C4	100.54 (7)	C7—Ru2—Ru3—C11	113.76 (10)
C2—Ru1—Ru2—C6	113.18 (10)	C4—Ru2—Ru3—C11	−152.01 (9)
C3—Ru1—Ru2—C6	−152.88 (10)	C6—Ru2—Ru3—C11	24.07 (10)
C1—Ru1—Ru2—C6	22.25 (10)	Ru1—Ru2—Ru3—C11	−75.70 (6)
P1—Ru1—Ru2—C6	−56.11 (9)	C5—Ru2—Ru3—Ru1	24.24 (19)
Ru3—Ru1—Ru2—C6	−76.82 (7)	C7—Ru2—Ru3—Ru1	−170.54 (7)
C2—Ru1—Ru2—Ru3	−170.00 (7)	C4—Ru2—Ru3—Ru1	−76.31 (7)
C3—Ru1—Ru2—Ru3	−76.06 (7)	C6—Ru2—Ru3—Ru1	99.77 (7)
C1—Ru1—Ru2—Ru3	99.07 (6)	C2—Ru1—P1—O13	−130.84 (10)
P1—Ru1—Ru2—Ru3	20.71 (5)	C3—Ru1—P1—O13	136.31 (10)
C2—Ru1—Ru3—C9	−145.78 (17)	C1—Ru1—P1—O13	−39.24 (10)
C3—Ru1—Ru3—C9	−67.89 (10)	Ru2—Ru1—P1—O13	38.22 (10)
C1—Ru1—Ru3—C9	109.23 (10)	Ru3—Ru1—P1—O13	56.51 (8)
P1—Ru1—Ru3—C9	17.40 (8)	C2—Ru1—P1—O12	111.15 (10)
Ru2—Ru1—Ru3—C9	−169.42 (8)	C3—Ru1—P1—O12	18.30 (10)
C2—Ru1—Ru3—C10	50.4 (2)	C1—Ru1—P1—O12	−157.25 (10)
C3—Ru1—Ru3—C10	128.24 (19)	Ru2—Ru1—P1—O12	−79.79 (9)
C1—Ru1—Ru3—C10	−54.64 (19)	Ru3—Ru1—P1—O12	−61.50 (8)
P1—Ru1—Ru3—C10	−146.47 (17)	C2—Ru1—P1—O14	−7.90 (10)
Ru2—Ru1—Ru3—C10	26.71 (17)	C3—Ru1—P1—O14	−100.75 (10)
C2—Ru1—Ru3—C8	−52.38 (17)	C1—Ru1—P1—O14	83.71 (10)
C3—Ru1—Ru3—C8	25.50 (10)	Ru2—Ru1—P1—O14	161.16 (8)
C1—Ru1—Ru3—C8	−157.38 (10)	Ru3—Ru1—P1—O14	179.46 (8)
P1—Ru1—Ru3—C8	110.80 (8)	O13—P1—O12—C12	144.95 (18)
Ru2—Ru1—Ru3—C8	−76.02 (8)	O14—P1—O12—C12	36.5 (2)
C2—Ru1—Ru3—C11	125.36 (16)	Ru1—P1—O12—C12	−92.85 (18)
C3—Ru1—Ru3—C11	−156.75 (9)	O12—P1—O13—C14	−48.5 (2)
C1—Ru1—Ru3—C11	20.36 (9)	O14—P1—O13—C14	53.7 (2)
P1—Ru1—Ru3—C11	−71.46 (7)	Ru1—P1—O13—C14	−176.12 (17)
Ru2—Ru1—Ru3—C11	101.72 (7)	O13—P1—O14—C16	78.01 (19)
C2—Ru1—Ru3—Ru2	23.64 (15)	O12—P1—O14—C16	−176.48 (18)
C3—Ru1—Ru3—Ru2	101.53 (6)	Ru1—P1—O14—C16	−47.2 (2)
C1—Ru1—Ru3—Ru2	−81.36 (6)	P1—O12—C12—C13	158.87 (18)
P1—Ru1—Ru3—Ru2	−173.181 (16)	P1—O13—C14—C15	109.1 (2)
C5—Ru2—Ru3—C9	53.2 (3)	P1—O14—C16—C17	−139.24 (19)
C7—Ru2—Ru3—C9	−141.5 (2)		

### Hydrogen-bond geometry (Å, °)

**Table:**

*D*—H···*A*	*D*—H	H···*A*	*D*···*A*	*D*—H···*A*
C12—H12A···O2^i^	0.97	2.60	3.558 (3)	171

Symmetry codes: (i) −*x*, −*y*+1, −*z*.
